# Measuring emotions: schizophrenia and family across cultural boundaries

**DOI:** 10.1017/mdh.2025.10047

**Published:** 2026-01

**Authors:** Ana Antić

**Affiliations:** Centre for Culture and the Mind, University of Copenhagen, Copenhagen, Denmark

**Keywords:** History of psychiatry, Transcultural psychiatry, Schizophrenia, Family, Expressed emotion, Decolonisation

## Abstract

This article explores how psychiatrists conceptualised the role of family relations and emotional atmospheres in the context of schizophrenia research in the second half of the twentieth century. It traces how families became the primary site to be mined and measured to explain schizophrenia’s onset, course and outcome, and zooms in on global psychiatric investigations of expressed emotion in families of schizophrenic patients, which aimed to offer a theoretical framework for understanding one of the most intriguing and influential findings of transcultural psychiatry: that schizophrenia appeared to have a shorter course and favourable recovery rates outside the Western world. The article engages with a wealth of research materials from schizophrenia and expressed emotion studies, and a variety of voices – clinicians, patients, families – which shaped these investigations. It also draws a comparison between this discussion of ‘traditional’ families as a beneficial environment for schizophrenia, and critical psychiatric and psychoanalytic discourses from the middle decades of the century which focused on the reportedly extreme psychopathological potential of ‘schizophrenogenic’ family relations in the Western world. Analyzed through this prism, expressed emotion research constructed the Global South as a preferable, even romanticized, alternative to the Western model of family interaction. On closer inspection, however, this idealization of the traditional family involved a variety of essentializing and romanticizing ideas which reinforced the ever-present binary of the modern West versus backward Global South, and perpetuated the belief in the decolonising and developing world’s cultural and intellectual simplicity.

## Introduction

‘We now have evidence that the prognosis for schizophrenia is much better in nonindustrial than in industrial societies’, wrote anthropologist Nancy Waxler in 1979, in the introduction to her seminal research on the relationship between cultural contexts and recovery from schizophrenia in Sri Lanka.^1^ Waxler’s article covered schizophrenic patients in Sri Lanka’s peasant communities and their clinical situation and social reintegration after five years. As Waxler reported, their five-year outcome was exceptionally good, and comparable to the results the International Pilot Study of Schizophrenia (IPSS) researchers recorded for patient samples in India and Nigeria, but much better than five-year recovery rates in developed countries such as Denmark, the UK, or even the USSR. In her analysis, Waxler focused on the importance of family norms, values, and relations as some of the most important explanatory factors for this notable difference between the course of schizophrenia in developed and developing countries.

Waxler’s influential arguments spoke to some of the most significant psychiatric research questions of the second half of the twentieth century: How to conceptualise the relationship between severe mental illness and cultural context, and what was the role of family relations and communication patterns in the onset and development of schizophrenia? Psychiatric investigations of family and its possible pathogenic potentials have had a long history in Western medicine – starting as early as the 1930s, psychiatrists, psychotherapists, and anti-psychiatrists singled out family environment as one of the most vital contexts for understanding schizophrenia and its origins.^2^ While the theory of schizophrenogenic mothers was rather short-lived, the focus on families was not, and family discussions and investigations played a major role in global psychiatric debates on schizophrenia and mental illness in the post-colonial period.

This article explores the complex history of these debates as they related to cross-cultural research in global psychiatry, and argues that family became a crucial site for understanding the relationship between the West and the decolonising world, and for establishing the universality of mental disorders in the aftermath of racist colonial psychiatry. Psychiatric discussions of family, culture, and schizophrenia opened up questions well beyond the field of mental health and shed light on how global psychiatry imagined and re-imagined the decolonising world and its core social and cultural traits in the aftermath of colonialism. This article aims to investigate the fundamental assumptions regarding schizophrenia, modernity, and ‘civilisation’ on which such discussions rested, and which can serve as a window into complex continuities and ruptures between colonial and post-colonial psychiatry. Moreover, debates and observations about families and their pathogenic potentials also reflected broader social and political concerns and were also often used to criticise what psychiatrists considered harmful trends in Western families. In that sense, as the article argues, global psychiatric research into family and schizophrenia was not fundamentally different from earlier theories of schizophrenogenic mother, which were deeply rooted in Western psychiatric, political, and cultural disputes.

In her piece, which opened this article, Waxler introduced some of the most important terms of this larger debate on the relationship between schizophrenia, family, and modernity. She argued that Sri Lankan families, which she described as comparatively straightforward, positive, and tolerant, crucially contributed to ameliorating the nature and length of schizophrenia in that society, because they were large and ‘strong’, which ‘[made] care of a sick person easier and [prevented] crisis and rejection.’ Moreover, such families provided their ill members with ‘positive social support’, which was ‘almost universally available’ in the Sinhalese society.^3^ Due to the ‘peasant norm of traditional society’, Waxler argued categorically that mentally ill patients in Sri Lanka never experienced isolation, rejection, and abandonment by families, and people from rural settings generally believed that ‘family must care for each other’.^4^ She then juxtaposed this with a much more individualised and isolated experience of schizophrenia in the West, where rejection, abandonment, or lack of family contact would be much more common.

One of the most important aspects of such families’ benign influence, according to Waxler, was their ‘traditional’ beliefs about mental illness, and the relatively low importance of medical discourses of schizophrenia in developing societies: ‘in many peasant societies … severe psychoses are believed to be brief, curable and leaving no lasting aftereffects’. In the case of Sinhalese patients, traditional healers would communicate this clearly and often even predict the exact date of the final recovery.^5^ In that way, it was socially expected – and predicted – that schizophrenias, psychoses, and other mental illnesses were to be of relatively short duration; and patients and their families rarely had access to the Western psychiatric paradigm according to which schizophrenia was a life-long and profoundly life-altering illness. Another crucial aspect of this traditional discourse was that it externalised the cause of illness – ‘a belief that mental illness is caused by supernatural agents who often act in an irrationally vindictive way, attacking persons without reason’ – and removed responsibility from the patient/sufferer.^6^ In Waxler’s Sinhalese examples, such magical, non-scientific beliefs of ‘pre-modern’ communities helped relieve schizophrenic patients of significant burden as well as guilt, because their illness was dissociated from any negative past experiences, moral failures, or physical/neurological defects.

This line of argumentation was crucially linked to the research results of the International Pilot Study of Schizophrenia, the most ambitious international schizophrenia study in the twentieth century, which involved nine research centres around the world in order to develop universal criteria and instruments for diagnosing schizophrenia in different cultural settings. One of the IPSS’s most intriguing findings was that, while the incidence of schizophrenia was comparable across cultures, its prognosis and outcome were significantly better in the research centres located in the developing world (in the case of the IPSS, these were Nigeria, India, and Colombia).^7^

This article will centrally engage with these assumptions and their implications. How was this global research on the relationship between families and schizophrenia conducted, and how did the explanatory framework of benign (and conflict-free) traditional versus schizophrenogenic modern families come to dominate the cross-cultural schizophrenia debate?

Following a brief introduction of the main findings and assumptions of the IPSS and its WHO-coordinated follow-up, Determinants of Outcome of Severe Mental Disorders Study (DOSMED), the article will explore the British and European origins of the core methods and research instruments used to measure the emotional index and atmospheres within families of schizophrenic patients. Relying on the archives of both the IPSS and DOSMED, particularly its sub-study on emotional relations and interactions in families, it will then move on to analyse how this family-focused schizophrenia research became internationalised, and which interpretive frameworks were utilised when the original instruments and concepts were applied to families from outside the Western world. The article examines how schizophrenia researchers adjusted their measurements and assumptions in order to explain the role of cultural differences and specificities in the context of family relations. Another important aspect of the argument pertains to the relationship between the schizophrenia family studies starting in the 1960s and the earlier psychoanalytically inflected research on ‘schizophrenogenic mothers’. The article interrogates the lines of continuity between these two bodies of knowledge, demonstrating that some of the core assumptions and findings of the latter were perpetuated and confirmed in the former. This is particularly important for understanding how global schizophrenia and family research romanticised ‘traditional’ families from the developing world while retaining a harsh, critical stance towards Western families and parents, which it inherited from the schizophrenogenic motherhood paradigm. While the research on schizophrenia and family was focused on the course and outcome of the illness and went to great pains to distance itself from psychoanalysis, the theories on schizophrenogenic motherhood were primarily driven by questions and assumptions about aetiology. However, the article demonstrates that the studies on expressed emotion also sought to look for the origins of schizophrenia in familial emotional interactions and parental behaviour, thereby broadening the scope of their own research questions and aims, and reinforcing their (unacknowledged) links to the theories of schizophrenogenic motherhood. Following a brief examination of the role of aetiology, the article ends by exploring the voices of those who were the main objects of these different strands of research – family members of schizophrenic patients. This segment engages with the archives of associations of families of schizophrenic patients, and highlights their responses, concerns, and critiques, demonstrating that the influence of the schizophrenogenic motherhood research heritage was indeed very visible at the time. Moreover, this part of the article emphasises the extreme importance of the processes of psychiatric deinstitutionalisation and their multifaceted effects on psychiatric care for understanding the development of schizophrenia and family research. This remains a vital aspect of the historical context, which schizophrenia researchers and mental health practitioners often omitted from their clinical accounts, but it was absolutely central to relatives’ own interpretations of how family was conceptualised within this body of knowledge.

In recent years, researchers have increasingly explored the concept of schizophrenia from historical, literary, artistic, and anthropological perspectives.^8^ These accounts produced invaluable insights into the meaning of schizophrenia inside and outside the clinic, shedding light on how the diagnosis was constructed, debated, and re-defined in different historical and political contexts. They have primarily focused on the Global North, and this article responds to the critical need in the historiography of schizophrenia and psychiatry to examine how this diagnosis was transformed into a global and transcultural category. It traces the complex history of post-colonial psychiatric efforts to understand and re-imagine how ‘non-Western’ cultural contexts interacted with the experience, onset, and outcome of the illness. Moreover, while the role of family in schizophrenia has been covered at length in psychiatric literature,^9^ historians have rarely engaged with schizophrenia and family research and its implications for understanding broader social and cultural histories of the psychiatric profession.^10^ The article offers an original account of how different bodies of knowledge about schizophrenia and family evolved and interacted with historical and political circumstances, and how they reflected wider concerns of the second half of the twentieth century – colonialism and decolonisation, as well as the effects of deinstitutionalisation on schizophrenia patients and their relatives.^11^

The relationship between schizophrenia and culture was an important topic for colonial psychiatrists. Colonial psychiatry posited that the incidence of mental illness, including psychosis, was significantly lower in colonised non-European societies. Leading British and French psychiatrists viewed the lower rate of psychological illness as a result of a simpler, less sophisticated neurological and psychological structure of non-European minds (according to John Colin Carothers, ‘African minds’ were comparable to those of ‘leucotomised Europeans’, so that their reported lack of individuation and psychological differentiation saved them from complex mental disorders, and from complex psychoses and schizophrenia in particular).^12^ These colonial arguments insisted on the simplicity of subjectivity formation and emotional relations in traditional societies. This line of thinking was decidedly evolutionary and hierarchical, comparing colonial subjects’ psychological and emotional capacities unfavourably to those of average Europeans. Some of the most prominent colonial-era psychiatrists (even if they belonged to the liberal group) declared non-Europeans to be insufficiently differentiated individuals, too reliant on their respective collectives (‘tribes’), and emotionally unbalanced.^13^ In the post-colonial period, early transcultural psychiatry often reiterated similar arguments, couching them in more positive terms: Canadian psychiatrist and one of the founders of the McGill school of transcultural psychiatry, H.B.M. Murphy, argued that schizophrenia might be the price societies paid for ‘civilisation’ and that it was significantly more prevalent in those communities marked by greater social and cultural complexity. Conversely, ‘traditional’ societies protected their members from schizophrenia because they were characterised by simplicity – of social, political as well as familial relations and organisation.^14^

In that sense, the IPSS and its findings regarding the universal cross-cultural incidence of schizophrenia fundamentally redirected the global discussion but offered an alternative argument, which might have rested on similar assumptions: While schizophrenia was not rarer in ‘traditional’ societies, it was a shorter and more benign illness. The IPSS set an agenda for an entire generation of schizophrenia researchers. The 1970s and 1980s saw a wide variety of researchers embarking on psychiatric and anthropological investigations in order to determine whether there existed specific cultural or biological factors that made schizophrenia a more benign, curable, and short-lived illness in the developing world.

In the course of these complex epidemiological projects, family relations, values, and attitudes emerged as one of the most promising and productive fields of examination. Indian psychiatrists and IPSS researchers Dube and Kumar emphasised the remarkably positive outcomes of schizophrenia patients from the Agra research centre and explained these cross-cultural differences with reference to particularly benign effects of traditional Indian family setups. Here again, the assumed importance of large supportive families came to the fore: ‘Subjects in this region get good family support and there are few demands on them … The fact that in India few families are nuclear may be another reason for better outcome. Also, there may be coping behaviors on the part of both family members and the subject’.^15^ In their own internal review, the UK Medical Research Centre also reported on this hypothesis, noting its relevance for schizophrenia studies and family-focused interventions among the British population: commenting on the IPSS, the MRC’s Social Psychiatry Unit speculated that ‘it is possible … that the extended family system common in many developing countries might result in a dilution of highly charged emotional relationships’.^16^ These arguments about family rested on a variety of culturally informed assumptions, which were nevertheless very rarely questioned or scrutinised. They often constructed a romanticised (and essentialised) image of a ‘traditional family’, characterised by simplicity and free of complex emotional relations and ambivalence, and set it up in striking contrast to the ‘modern’ model of family relations (typically encountered in the West and reportedly conducive to alienation and rejection).

Among the numerous schizophrenia chronicity studies in the second half of the twentieth century, DOSMED was again the most ambitious and wide-ranging in terms of both methodological sophistication and geographical reach. DOSMED increased and diversified the original IPSS network to twelve field research centres in ten countries.^17^ DOSMED was exclusively focused on investigating and interpreting differences in the prognosis of schizophrenia between developing and developed countries, and its core study consisted of clinical assessments and first- and second-year follow-ups. At the same time, four sub-studies were organised around four different areas, both social and clinical, which were hypothesised to play a role in determining the course and outcome of schizophrenia in different cultural contexts. One of the most influential sub-studies revolved around the role of family relations and patterns of emotional expression within families, exploring the ‘relationship between emotional interactions in the family and short-term prognosis of schizophrenia’.^18^ In order to measure the precise effects of emotional atmospheres and communication models in families, this sub-study set out to achieve what often appeared impossible – to quantify family life itself and its complex emotional exchanges, behaviours, and expressions, and to translate them into a coherent cross-cultural clinical instrument. Luckily, a group of UK psychiatrists and sociologists had already introduced the concept of expressed emotion (EE) in families in the 1950s and 1960s, and conducted a series of interviews to explore the relationship between the clinical status of patients diagnosed with schizophrenia and high levels of emotional expression in their family members.^19^ DOSMED’s sub-study of emotional interaction in families rested crucially on the concept of EE and related questionnaires and interview protocols, but it aimed to apply them beyond the Anglo-American research sample. It was conducted in the field research centres in Chandigarh (India) – which served as a representative of developing world cultures – and Aarhus (Denmark) – which was deemed a sufficiently exemplary Western cultural setting and comparable to the UK, where previous EE studies had been done. DOSMED researchers also hypothesised that ‘India provide[d] a marked contrast’ to Denmark, which made the choice of research centres appropriate.^20^

It was striking how much focus DOSMED placed on collecting, measuring, and analysing social, cultural, and family information. Ultimately, according to its final report, it did not reach significantly different results: it is concluded that ‘patients in the developing countries show a more favourable evolution than their counterparts in the developed countries’ and that ‘a strong case can be made for a real pervasive influence of a powerful factor which can be referred to as “culture”’.^21^ As in the IPSS, the concept of culture remained open and vaguely defined despite these declarations of its possibly far-reaching significance. But DOSMED did go some way towards specifying what culture might mean in the context of the study’s parameters, even if only indirectly. Family relations and the nature and degree of family members’ emotional involvement emerged as one of the most important areas of research, and this focus on families went some way towards developing a more substantial definition of cultural factors.

## Expressed emotions research

The concepts and measurements related to EE were not originally developed with cross-cultural contexts in mind, and the idea of culture played little role in shaping the broader theoretical framework of this research field. Research into EE within families was first initiated and implemented in the UK, in the context of investigating whether release from hospitals and return to families might affect the relapse rate of chronic schizophrenia patients. The original studies were conducted by an interdisciplinary team including George Brown, Morris Carstairs, and their collaborators at London’s MRC Social Psychiatry Unit, and ran between 1956 and 1972, and sought to explore the issue of hospital readmission of psychiatric patients.^22^ These studies identified the clinical significance of ‘high emotional involvement’ by parents and spouses of schizophrenic patients, but the concept of expressed emotion was a bit longer in coming. At first, the UK psychiatrists wanted to investigate whether patients who left psychiatric hospitals to live with their spouses or parents had a better chance of remaining healthy and avoiding the recurrence of psychotic episodes. As it happened, the opposite was true, and patients who went to live with families as opposed to independent accommodation experienced a higher relapse rate.^23^ Perhaps unsurprisingly, given the broader context of mid-twentieth-century schizophrenia research, relationships to mothers turned out to be particularly significant: It appeared that the less contact patients had with their mothers, the likelier they were to remain in remission so that patients whose mothers worked outside the house were doing better than others.^24^

Over time, the idea crystallised that ‘highly emotionally involved homes’ had a measurable negative effect on the course and outcome of schizophrenic illness, and researchers at the MRC Social Psychiatry Unit worked to identify specific aspects or components of the home emotional atmosphere which might be clinically relevant. These were at first adapted from research instruments that aimed to assess the impact of parental emotional expression on patients diagnosed with neurosis, and their causal relationship to relapse was further tested and rated. The final definition of the EE clinical construct settled on five components measured to have a significant impact on the outcome of schizophrenia: criticism, hostility, emotional overinvolvement, warmth, and positive remarks.^25^ As George Brown recounted, the expressed emotion research on families of schizophrenic patients produced one important addition, compared to the scales developed for neurotic patients: the concept of ‘emotional overconcern’ or ‘overinvolvement’ was deemed to have a particularly notable influence.^26^ In subsequent developments and applications of the EE construct and scales, emotional overinvolvement assumed tremendous importance as one of the most significant factors contributing to the chronicity of schizophrenia. In one of the paradigmatic examples of overinvolvement, a parent reported on their excessively self-sacrificing behaviour focused on the patient: ‘I’ve sacrificed my life for that child – anything she’s ever wanted, I’ve always tried to give her. My only concern has been her happiness, her well-being’.^27^ Another mother expressed a similar sentiment – inability to concentrate on other aspects of her life when faced with her child’s severe psychological illness: ‘But how can I think of anything else when my baby is locked up like that’?^28^ Overly critical and hostile attitudes were deemed equally important: EE researchers assessed both the tone and content of critical and hostile remarks, and focused on those comments and statements that indicated a generalised rejection of the patient’s person and behaviour as a whole.^29^ For instance, one father said, ‘He annoys me just by being there. I wish he’d walk out the door and leave us in peace’, while another parent described the patient in devastating terms: ‘He’s such a selfish person – he’s got no interest in anyone but himself’.^30^

Soon after these initial EE studies were conceptualised and completed, Julian Leff became involved in further investigations on British and US families, and his and Christine Vaughn’s research popularised the concept of EE and related methodologies. Leff was also a vital researcher not only in the IPSS but also in DOSMED and became one of the core architects of DOSMED’s efforts to internationalise EE measurements as predictors of the course and outcome of schizophrenia.^31^ DOSMED then adapted the existing interview protocols and rating scales, and the entire sub-study on emotional interaction within families rested on investigating the different components of EE in north Indian and Danish cultural settings. In the decades following DOSMED, clinicians and researchers developed and implemented hundreds of studies, which examined the relationship between family members’ behaviour and schizophrenia, and relied on EE assessments and measurements. In these investigations, ‘EE has emerged as the main predictor of relapse, regardless of the language and culture in which studies were conducted’.^32^ According to Leff, this reinforced the universalist framework of transcultural psychiatry and the WHO schizophrenia studies themselves: ‘the cross-cultural applicability of EE indicates that there must be a common basis for the expression of human emotions throughout the world’.^33^

Within the universalist framework of emotional expression, the EE index was found to differ substantially among cultures, and families in different cultural settings were reportedly marked by widely varying emotional indices. In the DOSMED series of investigations, British, Danish, and Indian family members of schizophrenic patients were compared, and the Indian sample had the lowest proportion of relatives with high EE ratings. While over half of Danish and British families were rated high on the EE scale, the corresponding number in Chandigarh was only 23%.^34^ In their analysis of the expressed emotions research results from the Chandigarh research centre, Leff and Indian psychiatrists Narendra Wig and A. Gosh repeated quite a few of Nancy Waxler’s core arguments. They insisted on the significance of traditional families’ non-medical understanding of schizophrenia as a ‘serious, legitimate illness outside of the patient’s locus of control’. Moreover, as in Sri Lanka, it was the relative rarity of small nuclear families that was considered central: ‘large kin-based households and networks in which a sense of the importance of family bonds induces relatives to assume responsibility for the patient’s care and recovery’. Finally, Indian relatives’ responses demonstrated superior coping mechanisms, which ‘[avoided] arguments and confrontation’.^35^ High EE relatives’ behaviour was confrontational in nature: They were unable to change course and adjust, and this often led to explosive and stressful encounters. On the other hand, low EE relatives (particularly those in the developing world) were ‘much more flexible and resourceful’, managing to de-escalate and sidestep potential conflicts.^36^ This image of conflict-free families in the developing world was surprisingly enduring and influential in the context of transcultural schizophrenia studies.

But underlying these different assumptions was one core interpretation of the relationship between culture and emotional expression, which was fundamentally informed by beliefs in the exceptional nature of Western individuality. This speculation about Western cultures’ unique historical focus on the development of the individual and introspection posited that modern Western societies produced more nuanced and differentiated personalities and forms of thinking and feeling, especially when compared to ‘traditional’ societies and their commitment to the group.^37^ Such ideas played a very important role in transcultural psychiatric discussions in the second half of the twentieth century. In the context of DOSMED and EE research, this interpretive framework indicated that Western families expressed feelings in a much more ‘untrammelled’ way precisely because of the broader cultural emphasis on self-reflection and on understanding/differentiating internal psychological states. As a result, the argument went, Western relatives of schizophrenic patients tended to have an increased awareness of their different emotions, and were more likely to be willing to talk about them.^38^ At the same time, the somatisation thesis sometimes made an appearance as well: ‘in many non-Western cultures emotional distress tends to be more readily expressed in somatic rather than psychological terms’; consequently, individuals from the developing world – and especially in rural families – were less likely to express their emotions in an open and unrestrained manner.^39^

DOSMED researchers zoomed in on ‘tolerant and accepting attitudes of family members’ and argued that the course and outcome of schizophrenia in India could be more benign because relatives indicated a ‘remarkable tolerance for both positive and negative symptoms of schizophrenia’, as well as having lower expectations, especially in rural settings.^40^ This was reflected in a peculiar and unique trend noted among relatives in the Chandigarh sample: a rapid drop in the overall Expressed Emotions index, as a result of a striking reduction in the number of critical and hostile comments, between the initial study interview and the first-year follow-up. As Leff reported, this was significantly different than in any Anglo-American sample of families, and exemplified Indian relatives’ exceptional coping mechanisms – their ability to become more accepting of the illness and its negative symptoms, and to devise appropriate strategies for handling and understanding different forms of pathological behaviour.^41^ As noted above, such interpretations were informed by a particular model of family relations and communication – in contrast to the UK and US examples, the Chandigarh social and clinical landscape was dominated by large, tolerant and supportive families, who rarely abandoned their ill relatives, and continued to live with and care for them despite their sometimes extremely difficult and disruptive symptoms – importantly, such families were ‘more characteristic of traditional rural societies than of industrial urban ones’.^42^ And indeed, according to the DOSMED results for Chandigarh, rural families fared much better on the EE scale: Only 8% of rural relatives demonstrated high EE, as compared to 30% of city-dwelling family members. At the first annual follow-up, ‘the urban relapse rate was 19% compared with a rural relapse rate of 9%’.^43^ Moreover, even if they demonstrated no magical or anti-medical thinking, Indian relatives still insisted that the patients should bear no responsibility for their behaviour: ‘The relatives concerned seem to have accepted that the patients suffer from an illness and consequently no longer blame them for difficult behaviour’.^44^ In fact, out of forty interviewed relatives, only four assigned any degree of blame and responsibility to the patients, and Leff, Gosh, and Wig concluded that the attitude towards responsibility was crucial to understanding both family members’ levels of hostility/criticism and the outcome of schizophrenia in a particular culture.^45^

The Chandigarh research sample reportedly had a lot in common with Mexican-American families of schizophrenic patients, whose involvement and responses were analysed in significant detail from the 1980s on. In 1987, Marvin Karno and his collaborators conducted an investigation, which confirmed the earlier British and North American findings. Those patients from Mexican-descent families whose key relatives were deemed to express a high level of emotional involvement, criticism, and hostility had significantly increased chances of relapse.^46^ Moreover, researchers argued that, in this socio-cultural setting, a significantly smaller number of family members scored high on the EE scale: Compared with 67% of Anglo-American households, only 41% of Mexican homes were categorised as ‘high’ in terms of relatives’ emotional involvement.^47^ The comparison was particularly striking when it came to expressions of criticism and hostility, as ‘Mexican-American family members [appeared] to be far less critical of their ill relatives than their Anglo-American counterparts’.^48^ This was confirmed in another study, which concluded that a significant majority of the Mexican-American sample (71.6%) was not ‘critical, hostile or intrusively involved with the patient’.^49^ Karno’s research focused on ‘low-income, relatively unacculturated’ families – as we will see below, other work on Mexican-American families confirmed that lower socio-economic status, rural background, and little formal education tended to decrease EE scores, and it was precisely these more ‘traditional’ familial and cultural settings that seemed to provide an atmosphere conducive to recovery from schizophrenia.^50^ Notably, these studies identified pockets of ‘traditional culture’ embedded in the developed world and aimed to show that these communities’ language and values crucially shaped their members’ experience and outcome of schizophrenia – setting those patients apart from common trends in the dominant culture.

Anthropologist Janis Jenkins subsequently took the lead in the field of EE studies in Mexican-American communities – Jenkins’ research confirmed that large and supportive Mexican-American families had a beneficial effect on the duration and curability of their relatives’ schizophrenia. Here again, culturally inflected familial conceptualisations of illness assumed great interpretive significance, and Jenkins argued that Mexican Americans’ distinctive conceptualisation of schizophrenia played a large role in shaping their comparatively more inclusive and sympathetic attitudes to ill family members.^51^ In the Anglo-American cohort, relatives predominantly relied on the medical framework to understand the nature of schizophrenia and its implications: They adopted the psychiatric language of ‘biochemical imbalances’ and biological factors, which aimed to reduce stigma and remove personal responsibility, but, in Jenkins’ interpretation, produced much more ambiguous results. The medical disease model, to which most Anglo-American respondents seemed to subscribe, did not necessarily eliminate the tendency to blame the ill person for their behaviour, nor did it prevent family members from focusing on and criticising personal traits and attributes. Given the overall cultural context, the existence of a ‘legitimate’ illness did not mean that patients were not seen to have some degree of individual responsibility for acting against it.^52^

In line with this, Jenkins reported that, despite their acceptance of the medical framework, families also often insisted on describing their relatives’ negative personal characteristics (such as ‘laziness’) when they tried to make sense of schizophrenia. On the other hand, Mexican families used ‘nervios’ as a ‘cultural label’ to describe what went wrong with their schizophrenic children or spouses, and this broad concept provided a platform for a much more inclusive as well as sympathetic interpretation of schizophrenia. ‘Nervios’ could encompass a large variety of psychological disorders and experiences – severe psychotic illnesses as well as milder forms of distress over life’s hardships – so that it functioned as a buffer against othering.^53^ While in British or Anglo-American contexts, the diagnosis of schizophrenia could be deeply alienating as it often meant that the patient exhibited a variety of bizarre and seemingly incomprehensible behaviours, Mexican-American family members could recall their own previous bouts of ‘nervios’ as a shared experience, an affective bond with the schizophrenic relative that made it possible to understand – at least partially – what they were going through. This enabled ‘the maintenance of close identification of family members’.^54^ At the same time, nervios appeared to remove personal responsibility much more straightforwardly and efficiently than mainstream medical categories. The word connoted precisely the opposite – ‘the individual’s loss of control in the face of difficult life circumstances’.^55^ In contrast, Anglo-American families’ familiarisation with scientific language and paradigms seemed to do them and their ill relatives little good. They were notably more hostile and unempathetic, and ‘more likely to be matter-of-fact or vitriolic in their descriptions of the illness’.^56^ Jenkins explained that the Mexican Americans in her study did not have much access to the psychiatric/medical conceptualisations of schizophrenia – mainly because little medical information was available in Spanish, but also, presumably, due to her informants’ low educational levels. Jenkins thus framed this absence of medical knowledge and understanding as a significant positive factor aiding recovery.^57^ These families’ lack of familiarity with medical research, and their extremely limited understanding of medical categories, procedures, and prognoses in fact exerted a positive influence on their relatives’ course of illness and chances of recovery. It was not entirely clear from this research how exactly this happened, but the argument seemed to suggest that this non-familiarity with scientific paradigms acted as a sort of self-fulfilling prophecy. If families and patients did not know that schizophrenia was a difficult and life-long illness, it actually became less complex and more curable.

At the same time, anthropological research with Mexican-American families and schizophrenia brought forward some major criticisms of how the EE scales were used and conceptualised cross-culturally. Karno and Jenkins have referred to the EE construct as a ‘prediction without meaning’, arguing that researchers demonstrated a close correlation between EE index and relapse rates but were less interested in analysing the nature and content of expressed emotion categories in different social and cultural contexts, and in developing their broader theoretical framing.^58^ Despite the criticism, however, this research further reaffirmed the cross-cultural applicability and the relevance of the core components of EE to the course and relapse rates of schizophrenia across the world.

Global psychiatric research on expressed emotion in families thus zoomed in on two core qualities of traditional families, which were deemed particularly salient: the nature of family ties and relationships, and their ideas about mental illness and its causality. In these contributions, which remained exceptionally influential in psychiatric understandings of schizophrenia at the end of the twentieth century, the romanticised concept of traditional family (and its assumed universal supportiveness) was combined with the notion that ‘traditional’ societies relied on a fundamentally different interpretive framework of schizophrenia – presumably one that did away with notions of personal responsibility and (moral) guilt (this was one of the core arguments of Murphy and his colonial predecessors – that non-Western communities lacked in precisely those concepts and rarely expected their members to take responsibility for their behaviour or act in an autonomous way^59^). And unsurprisingly, higher levels of expressed emotion, with their attendant harmful effects on the course and outcome of schizophrenia, were measured in urban (modern, developed, more Westernised) areas of the developing world, while rural families reportedly remained closer to the traditional norm psychiatric researchers imagined, and were thus more beneficial for their schizophrenic relatives. As in the earlier psychiatric arguments about lower incidence of schizophrenia in the developing world, these debates about families and expressed emotion framed the difference in course and outcome in terms of traditional societies’ comparative advantages – families from the decolonising world received an almost exclusively favourable assessment and were set up as a valuable model, one that Western societies could and should learn from. However, on closer inspection, this idealisation of the traditional family involved a variety of essentialising and romanticising assumptions, which reinforced the ever-present binary of the modern West versus backward Global South, and perpetuated the belief in the decolonising and developing world’s cultural and intellectual simplicity. Moreover, this particular image of benign and conflict-free traditional families often tended to ignore or marginalise violence, hierarchies, and oppression that happened regularly within family settings across the world.

## Origins of the expressed emotions research

This idealisation and romanticisation of ‘traditional’ families contained within itself an implicit (and sometimes explicit) critique of Western family models and communication patterns. In that sense, it had a great deal in common with mid-twentieth-century schizophrenia research, as well as with the pre-DOSMED EE research conducted primarily on British and North American families. George Brown insisted that, in their original conceptualisation, investigations of EE and schizophrenia did not aim to put the burden of responsibility on family members. Brown consciously distanced himself from research on ‘schizophrenogenic mothers’, arguing that such theories overemphasised ‘enduring, deeply disturbed relationships’ and that ‘the presence of [such] unusual relationships between parents and patients should not be allowed to dominate our thinking’.^60^ Instead, the initial measurements of emotional atmospheres were much more focused on understanding the ‘ordinary’ and ‘commonplace’ in family behavioural patterns. Moreover, these hypotheses and conclusions were not about assigning blame: They assumed complex interactions between individual proclivities and family settings, and speculated that patients with schizophrenia might be particularly sensitive to emotional intensity and involvement. Most importantly, the pre-DOSMED EE research did not engage with questions of aetiology: Instead of proposing that certain kinds of pathological parent–child relations *caused* schizophrenia, they only explored how family members’ feelings and communication might affect its course and prognosis.^61^

However, EE research and earlier investigations on schizophrenogenic parents/families had quite a few common themes. For instance, EE explorations identified high levels of emotional involvement in British and American (‘Western’) families, and they judged this type of emotional atmosphere to be harmful for the recovery of schizophrenic patients. In an important way, this was a rather negative assessment of the Western family model. Studies by psychiatrists such as Leff and Brown were unusually categorical in their results. Brown’s studies indicated that ‘the risk of deterioration in clinical condition was increased when prolonged contact with close relatives in the house was unavoidable’.^62^ Out of 128 schizophrenic men that this study researched, more than a half deteriorated (most of them getting readmitted to the hospital at least once) in the course of a year following their release, and these relapses were all linked to harmful emotional interactions within family.^63^ Moreover, Leff’s and Vaughn’s 1976 investigation of the emotional index of relatives of British patients from three South East London hospitals offered further confirmation: It is concluded that ‘a high degree of emotion expressed by the relative at the time of key admission remains the best single predictor of symptomatic relapse during the nine months following discharge’ and that ‘two thirds of those who were socially withdrawn or avoided family members in the months preceding key admission were well at follow-up’.^64^ In other words, families with higher levels of emotional expression and intensity were deemed harmful for the recovery of schizophrenic patients, and British and American families were often found to suffer from precisely this kind of overinvolved, domineering, or hostile emotional behaviour.

Moreover, when EE researchers tried to probe the exact meaning of expressed emotions, their conceptualisations often focused on negative personal characteristics and attributes. For instance, Christine Vaughn and Leff concluded that, in high EE families, key members were likely to be very intrusive, could not control their emotions, such as anger, anxiety, and distress, and were intolerant, unsympathetic, and impatient.^65^ As we saw above, when psychiatric studies of schizophrenia started measuring the EE index in different national and cultural settings, primarily in the decolonising world, such perceptions of Western familial models of interaction as harmful came into a particularly sharp relief, as non-Western (‘traditional’) families fared much better on EE scales. Their overall levels of detrimental emotional involvement were reportedly much lower, which marked a fundamental distinction from British and American relatives – parents and mothers in particular.

In Brown’s early studies of schizophrenia and emotional involvement, one important component focused on dominant or ‘directive’ behaviour of a ‘key relative’, defined as ‘the most closely related female living in the household’, typically mother or wife.^66^ The focus was often on overly dominant and domineering behaviour by mothers – the figure of a mother which emerged from these case studies and clinical notes was not dissimilar from earlier psychiatrists Trude Tietze’s and Frieda Fromm-Reichmann’s descriptions of schizophrenogenic behaviours: overbearing, hostile, manipulative, overprotective.^67^ For instance, one family was deemed to exhibit ‘very marked and continuous dominant behaviour’ of a key relative to the patient because the mother ‘talked excitedly most of the time’ and ‘issued several orders to her son’. Another mother with ‘not marked but unmistakable’ dominant behaviour ‘watched [her son] closely all the time and once brushed cigarette ash off his shirt’.^68^

But the concept of schizophrenogenic mothers was not solely determined by its psychiatric and clinical contexts. As Carol Hartwell, Elaine Showalter, and other researchers have emphasised, the core negative qualities that psychiatric practitioners criticised in schizophrenogenic mothers reflected broader social, political, and cultural concerns regarding the changing role of women in the middle decades of the twentieth century.^69^ As US-based psychiatrist John Rosen noted in 1947, ‘a schizophrenic is always one who is reared by a woman who suffers from a perversion of the maternal instinct’ – in this interpretation, ‘schizophrenogenic’ maternal behaviours could all be traced to pathological changes in women’s ability and willingness to fulfil their ‘natural’ parental and nurturing roles. Rosen connected such ‘perversions’ to harmful cultural and societal changes, in the course of which women sought to adopt ever more masculine professions and forms of behaviour.^70^ Along similar lines, other researchers of the concept described schizophrenogenic mothers’ rejection of their traditional ‘homemaking role’, their high ambition (one of Theodore Lidz’s paradigmatic examples was a ‘former career woman from a wealthy family who hated housework’), and consequent domineering attitude.^71^ Female emancipation thus carried within itself the seed of schizophrenia – it ‘unmanned’ men and undermined women’s central role in maintaining healthy family relations. According to Lidz’s research, perversions of biologically assigned parental roles could lead to serious pathological developments, and deviations from these traditional roles were particularly harmful when children were involved: ‘a cold and unyielding mother will be more deleterious than a cold and unyielding father’ – presumably because the maternal role was ‘naturally’ nurturing and affectionate. On the other hand, ‘a weak and ineffectual mother’ would cause less damage than such a father, who needed to be strong and dominant.^72^

Moreover, according to Hartwell’s analysis, the original concept of schizophrenogenic mother emerged under the strong influence of David Levy’s description of ‘overprotective mothers’: in addition to being domineering, such a mother ‘impeded her child from becoming independent, infantilized him, was overinvolved, and exerted too much or too little control’.^73^ In the subsequent psychiatric and psychoanalytic debates on schizophrenogenic motherhood, both overprotection (‘overanxiousness’ and ‘oversolicitousness’) and rejection or emotional coldness played equally important roles. The idea of overprotection was not dissimilar from the core concept in the later EE research – emotional overinvolvement/overconcern.

Some psychiatrists believed that schizophrenogenic mothers – both domineering and emotionally suffocating – had a particularly harmful effect on their male children, whose independence and autonomy they regularly sought to undermine as they forced and manipulated their sons to submit to their will.^74^ This was an obvious consequence of overly ‘directive’ and dominant motherhood, but it was also a way for such mothers to fulfil their own ambitions in relation to men. EE researchers certainly described a large number of comparable cases and analysed different ways in which high EE parents (and especially mothers) undermined the autonomy of their sons. For instance, in 1976, Leff and Vaughn reported on a mother who scored high on the overinvolvement, criticism, and hostility scales, and demonstrated ‘many attempts to exert a controlling influence through her nagging and “If only you listened to me” advice’.^75^ On the other hand, the concept of schizophrenogenic motherhood also played a vital role in the 1960s anti-psychiatric debates on schizophrenia, but some of the leading practitioners of the movement, such as R.D. Laing or David Cooper, primarily focused on their female patients. These were daughters of oppressive patriarchal families, whose schizophrenic breakdowns Laing interpreted as a rational response to highly conflicting messages and expectations regarding ‘the female role’ and normative femininity.^76^ In that sense, family was the essential context for understanding the onset and course of schizophrenia: It was a social institution vital to implementing patriarchal values, posing demands on and disciplining young women, whose madness was a form of protest against their parents’ attempts to curb their independence and autonomy. Moreover, ‘Laingian theory interpreted female schizophrenia as the product of women’s repression and oppression within the family’, a state caused in itself by women’s ‘violation of sex-role expectations’.^77^ In other words, the notion of rejecting normative femininity played an important role in anti-psychiatric understandings of schizophrenia as well – except in this case, anti-psychiatry problematised the impulse to pathologise this rejection. Yet, in spite of this feminist conceptualisation, as Showalter has argued, anti-psychiatry still insisted on holding mothers responsible, and failed to offer a systematic analysis of the relationship between schizophrenia and the complex socio-political circumstances of womanhood.^78^ Most importantly, it did not probe deeper into possible misogynistic aspects of the theory of schizophrenogenic mothers.

In conclusion, just like in the EE research, schizophrenogenic mothers could combine extreme emotional coldness, distance, and aggression with overconcern and overinvolvement – these seemingly contradictory behaviours were brought together in a figure of a mother who rejected her traditional, nurturing, home- and family-making role. At the same time, EE researchers dropped all references to the negative effects of emancipation and ‘aggressive femininity’, but described high EE mothers and some of their core traits in strikingly similar terms.

## Return of causality: reconsidering the aetiology of schizophrenia

There was another area of investigation that connected EE research and the concept of schizophrenogenic motherhood. Despite Brown’s insistence that EE research had not in any way been conceptualised as a study of aetiology, questions and arguments about the relationship between high EE family environments and the onset of schizophrenia certainly found their way back into the discussion. Most importantly, by the 1980s EE researchers claimed that there was enough evidence to indicate that ‘overprotective and overinvolved parental attitudes often [developed] very early on in a child’s life,’ and this suggested that such relationships could have contributed to the first onset of schizophrenia.^79^ In many of their own case studies, for instance, Leff and Vaughn found confirmation that problematic parental behaviours such as persistent criticism and hostility, as well as exaggerated self-sacrificing and overly protective tendencies often had long histories in families of schizophrenic patients. Their research showed that high EE parents practiced precisely those kinds of emotional responses for years, sometimes even decades, before their child experienced the first episode of schizophrenia.

The research on EE and causality originally relied on follow-up studies from US child guidance clinics, which had collected much information on their client children’s family dynamics and environments since the 1920s and 1930s. Focusing on families of those children who were later diagnosed with and treated for schizophrenia, researchers identified emotional trends in parents’ behaviour which were also highly relevant as predictors of relapse according to the EE scales. These included overprotective behaviour which undermined children’s attempts to separate emotionally and achieve independence, and cold and withdrawn marital relations (‘emotional divorce’).^80^ In other words, these studies, conducted in the 1950s and 1960s, could document instances of such behaviour in preschizophrenic children, and argued that significantly higher levels of emotionally ‘overinvolved’ responses and significantly worse marital relations were observed in parents of those children who subsequently experienced severe psychiatric disorders, compared to control groups (parents of children who did not get the schizophrenia diagnosis later in life).^81^ This was a very intriguing finding from the perspective of EE research, primarily because it seemed to indicate that one core component of the EE construct – emotional overinvolvement – played an unusually significant role in the family dynamics and parental behaviour *before* the onset of schizophrenia. Could these attitudes and patterns of behaviour/communication have an additional explanatory value, and suggest something important about the origins and causes of schizophrenia?

Encouraged by these results, Leff and Vaughn analysed their own cases and identified many in which high-EE attitudes and responses predated the emergence of schizophrenia. As in the child guidance clinics examples, these mainly concerned what was considered emotional overconcern and exaggeration – overly devoted or attentive attitudes on the part of parents. In one family from LA, both parents were deemed very highly involved (they scored four out of five on the emotional overinvolvement scale) because they praised their child ‘extravagantly’ and demonstrated ‘some lack of objectivity’ as they often spoke very positively about their son’s academic achievements and talents before he had a schizophrenic breakdown. The son was reportedly a ‘model child’ before falling ill, and the parents seemed unable to overcome their sorrow over this tragic loss of an exceptional young man’s potential and future.^82^ In quite a few cases, Leff and Vaughn identified harmful overprotective behaviour in parents whose children had suffered major illnesses from an early age. In such situations, parents were criticised for their excessive dedication and ‘self-sacrificing’ attitudes, and for being preoccupied with their children’s physical and psychological vulnerabilities: one ‘overinvolved’ mother, for instance, ‘dwelled on her daughter’s childhood illnesses’,^83^ while another ‘responded to the patient’s failure to grow normally [Turner syndrome] by being overprotective’ and feeling guilty about it.^84^ In the case of a mother whose son developed a difficult chest infection as a very young child, Leff considered that her overprotectiveness was ‘aroused by a severe childhood illness’ but concluded that her attitude was likely ‘already oversolicitous and had merely crystallized around the illness’.^85^

In their analysis of the cases, Leff and Vaughn still attempted to distance themselves from simplistic interpretations, emphasising the complexity of potential etiological factors and noting that it was unlikely, as the earlier research on schizophrenogenic mothers might have suggested, that certain kinds of parental attitudes and behaviours produced schizophrenia in any straightforward manner. However, this etiological turn in the EE research was very significant, as it provided yet another parallel to the concept of schizophrenogenic parents. It demonstrated that EE researchers were willing to go beyond Brown’s original framework, which distanced itself from explorations of causes and conceptualised different forms of expressed emotion as family members’ responses to schizophrenia and schizophrenic behaviours. On the contrary, when it came to discussing etiological aspects of expressed emotion, psychiatrists such as Leff and Vaughn offered a novel perspective on this research field: They argued that high EE attitudes often predated schizophrenic breakdowns so that they were likely to have a significant impact not only on the course and relapse in schizophrenia but also on its onset.

## EE and model parenthood

EE researchers produced a wealth of material on schizophrenia patients, their parents, and spouses, with detailed descriptions of family members’ behaviour, responses, and personal characteristics. From these documents and psychiatric researchers’ comments and assessments, there emerged various positive and negative models of parenting, and EE researchers inadvertently drew normative images of mothers and fathers, whose attributes were declared to have a significant influence on schizophrenic patients’ clinical status and outcomes. In the context of schizophrenia, recovery, and relapse, what were the ideal mothers and fathers like, how were they meant to respond, and what individual characteristics and forms of behaviour were best avoided? Equally importantly, how did these models of good and bad parenthood interact with cultural contexts and assumptions?

Leading EE researchers insisted that ‘a non-intrusive, tolerant approach by relatives’ was clinically the most beneficial, and most conducive to recovery from schizophrenia.^86^ The full implications of this argument depended vitally on how intrusion and (emotional) intrusiveness were defined. Describing their British sample, Leff and Vaughn reflected that they had been ‘struck by the exceptionally calm and self-contained responses by low-EE relatives, sometimes in the face of extremely agitated or bizarre behaviours’.^87^ Moreover, these family members were particularly adept at ‘diffusing crises’, and were either ‘naturally easygoing’ (‘happy-go-lucky’, as one mother reportedly described herself) or taught themselves how not to express their own emotions of anxiety or upset.^88^ One mother who was deemed to exhibit no signs of overinvolvement ‘invariably dealt with the patient’s displays of highly disturbed behaviour in a calm and rational way’, and never behaved in ‘unusually self-sacrificing, devoted or overprotective’ ways. The mother was praised for her balanced attitude and ability to remain composed in the face of her daughter’s aggression and violence, but it was her setting of boundaries that seemed to win the psychiatrists’ ultimate approval: She was not prepared to allow her daughter to return to the household unless she fulfilled certain conditions, and she appeared unconcerned with her daughter’s major life choices.^89^ It was primarily this distance that was considered beneficial for the daughter’s recovery prospects, and also the mother’s (rather exceptional) ability not to react in an emotional way to what was described as highly disruptive behaviour. Was the mother genuinely unconcerned, or, alternatively, how did she deal with her unexpressed (suppressed?) feelings of anxiety, fear, or anger? This is something Leff and Vaughn rarely commented on. In a similar vein, the father of William was commended for his ‘composure’ when describing his son’s multiple suicide attempts (although the fact that he did react ‘emotionally’ when the attempts happened was held against him).^90^ The mother of Barry was assessed favourably for not getting involved in her son’s problems, even when he seemed to put his own grandmother in danger: ‘I’d say “Phone the police if it gets bad – what do you expect me to do about it?”’, she reportedly told the grandmother.^91^ Even though the mother of Gary was deemed overinvolved for other reasons, she gave one answer that convinced the psychiatrists that she did have the capacity to improve her EE score. As Gary was going in and out of hospital and was habitually verbally aggressive towards her, ‘she felt she should have sat down and talked more with [him] and find out what was going on in his life but she had “forty other things going on”, so she didn’t’.^92^ Here again, when a parent chose a distant attitude and indicated that the child was not a priority, the researchers described it as a desirable form of behaviour.

On the other hand, even those parents who were rated fairly low on the overinvolvement scale were often criticised for ‘overconcern’ when they appeared worried about or focused on their children’s health problems and behaviour. In the case of the parents of Mike, the perceived overconcern consisted of their preoccupation with the origins of Mike’s schizophrenia and their own possible responsibility in it, as well as their efforts to get him to talk about his problems and feelings. Their decision to spend a week with him in hospital was deemed ‘self-sacrificing’.^93^ The behaviour of the mother of Charles raised some psychiatric red flags when she confided that she worried a lot about her schizophrenic son – ‘he’s my only worry, really’^94^ – and other parents who articulated sadness or anxiety over their children’s illness were commonly rated higher for emotional overinvolvement: One mother became tearful when talking about her son’s difficulties and that reaction on its own scored her a two on the scale.^95^ While the psychiatrists commented positively on Gary’s mother and her reported lack of time to check in with Gary regularly, the relationship between Peter and his mother was described as ‘enmeshed’ because she would spend hours talking to him about his feelings and problems – in her own account, she was responding to a real need: ‘it felt as if he needed to do that, and nobody else would do that with him’.^96^

Leff and Vaughn made sure to differentiate this ability to control emotional reactions from denial mechanisms, but they also indicated that there were at times very powerful emotions at work and that relatives experienced extreme feelings of fear, anguish, and depression.^97^ It remained unclear what happened with these emotions, and how they were channelled in inter-personal and family contexts. Leff and Vaughn did note that ‘a cool response in crisis does not necessarily indicate a lack of emotion’; instead, they argued, low EE family members likely learned how to control their emotions as they had to cope with frequent outbreaks and episodes of difficult symptoms over the years.^98^ Still, the emphasis remained on emotional distance and restraint. Given the British researchers’ focus on lowering and controlling the expression of emotions, were ‘unexpressed emotions’ always beneficial for family dynamics? Hatfield et al. suggested that ‘high unexpressed emotion’ could result in withdrawal, apathy, and psychosomatic illnesses on the part of family members and could also fundamentally undermine their relationship with the patient.^99^

On the other hand, as we saw above, in the North Indian sample a significantly smaller number of relatives exhibited high levels of emotional overinvolvement, and Chandigarh families were commended for finding the right tone for engaging with schizophrenic patients – without emotional excesses, exaggerated dedication or self-sacrifice, but also without overt conflict and blaming. As per Vaughn’s interpretation, in the developing world relatives of schizophrenic patients ‘frequently [demonstrated] an ability to cope with crises effectively and appear to exert a calming influence’; they were also overall more respectful of patients’ need for individual autonomy and distance, and were significantly more tolerant of the symptoms of schizophrenia and its related disabilities, placing few expectations on their ill family member.^100^ In the follow-up interviews, a year after the original study, Indian relatives’ emotional attitudes towards their ill family members kept evolving in the right direction, and their behaviour was even more unlikely to score high on the EE scale. One mother shared that ‘I don’t get angry with her. I am satisfied that she has become alright after treatment’. A father described a change in his family’s perception of the patient: ‘Previously we also used to lose temper with him but now we talk to him with love and affection, and that this thing is good for you.’ Yet another relative reported that ‘now I have adjusted myself to him since I have come to know about his illness’.^101^ It was these statements and emotions that were emphasised as examples of positive behaviour on the part of a relative of a schizophrenic patient, and as particularly representative of the beneficial trend of decreasing harmful forms of emotional involvement among family members. They informed an influential and long-lasting alternative to the Western model of family relations and communication – one that was considered conducive to preventing relapse and lowering the risks of chronicity.

This raises the issue of warmth as another significant component of the EE index. In this section, I have argued that EE researchers put forward a certain form of emotional distance as a highly positive trait in family relations, at least in the British and North American contexts. But how did the concept of warmth then shape this particular understanding of harmful or beneficial inter-personal relations within families of schizophrenic patients? In fact, expressions of warmth as a core EE component were increasingly marginalised in this line of research. Leff noted that ‘warmth was found to indicate a good prognosis in the British studies but was not incorporated in the EE index because when linked with overinvolvement a bad outcome ensued’.^102^ This position was not surprising: As we saw, EE researchers often considered parental expressions of affection and care to increase the risk of relapse. In other words, it was more important to avoid ‘emotional overinvolvement’ than to encourage warm interpersonal relations. On the other hand, the Chandigarh sample demonstrated that families often combined high warmth and high criticism – ‘a combination that was not commonly encountered among Anglo-American families’^103^ – and that in those situations critical comments did not lead to higher rates of relapse. DOSMED researchers hypothesised that in Indian families warmth might have neutralised the harmful effects of criticism (and hostility) – an important concession to culturally conditioned forms of inter-personal relations and communication: ‘A European might well regard warmth and criticism as incompatible emotions, whereas North Indians appear to have no difficulty in expressing both about the same patient during the hour of the interview’.^104^ Here again, the concept of overinvolvement and its complex transcultural expressions played an important role: While warmth was often associated with overinvolvement in Western families, which fundamentally undermined its potential benefits, emotional overinvolvement was sufficiently rare among North Indian relatives that researchers considered reinstating warmth as a core component of the overall EE construct. However, the number of Indian relatives who were rated as highly critical was too low for any statistically reliable conclusions to be drawn regarding the emotionally protective properties of warmth in non-Western families.^105^

## Voices of families

Given that EE research developed such a widely influential critique of Western family models and argued that Western families produced emotional behaviours and atmospheres, which were particularly harmful to people trying to recover from schizophrenia, it is worth exploring how families themselves responded to this debate. In the Western world, relatives of schizophrenic patients joined the broader conversation about the clinical relevance of family practices and communication models and asked important questions regarding unspoken assumptions (about responsibility as well as the structure of the mental healthcare system), which guided EE research.

Unsurprisingly, some mental health researchers, as well as families of patients diagnosed with schizophrenia, worried that many assumptions inherent in the growing field of EE studies constituted a continuation of earlier psychiatric and psychoanalytic research, which placed sole responsibility for the emergence of schizophrenia on families and mothers. As Agnes Hatfield and her collaborators from the National Alliance for the Mentally Ill (NAMI), a US-based grassroots organisation founded by family members of people diagnosed with mental illness reported, ‘EE theory is not enough of a departure from traditional theories that blamed families for mental illness to overcome families’ feelings of alienation from the mental health profession’.^106^ One of their core concerns was that an exceptionally complex psychological and emotional experience was reduced to simplistic categories – as Hatfield et al. noted, the EE research design could only classify families as high or low, and could not possibly capture the nuanced and dynamic nature of familial responses to schizophrenia (for instance, it left no space for conceptualising emotional responses as a continuum). Instead, it held a lot of potential for ‘negative labelling’ and stereotyping of certain families as ‘bad’ if they were not coping well with, or were stressed by, their ill relatives’ diagnosis at any point during the long process of schizophrenia treatment.^107^ Despite Brown’s, Leff’s, and others’ nuanced attempts at distancing their own project from hypotheses about schizophrenogenic mothers, therefore, family members could not miss some major common themes and approaches linking the two bodies of research. As I already showed, one important area of continuity was the persistent assumption of causality – even though EE research did not seek to hold high EE families exclusively responsible for the onset of schizophrenia nor did it offer sufficient evidence to prove such a causal relationship, a significant number of researchers and practitioners in the field started making these broader connections, both directly and indirectly.

The NAMI’s response also insisted that there was a breakdown of trust and meaningful communication between families of schizophrenic individuals and the mental health profession at large. This breakdown was primarily reflected in what the authors perceived as psychiatric researchers’ tendency to focus on negative aspects of family behaviours and their ‘deficits’ instead of understanding fully the complexity of family members’ care for ill relatives, and their many unobserved efforts to support schizophrenic individuals through tremendous personal ordeals: ‘professionals show little understanding of how families experience mental illness, its tremendous burdens, and its terrible sorrows’.^108^

This was of direct relevance to families of chronically mentally ill patients and, in fact, became one of the core topics in their advocacy efforts. In the United Kingdom, families and carers of schizophrenia patients came together in the early 1970s to create a National Schizophrenia Fellowship, which insisted on drawing attention to the inadequate healthcare support services for the severely mentally ill, especially in the face of deinstitutionalisation, in the course of which ‘the chronically mentally ill [were] abandoned to their fate’ and released to vaguely defined (and often unreceptive) ‘community care’.^109^ ‘The explicit assumption accompanying the hospital run-down, and the present proposals for closures’, the NSF argued in 1983, ‘has been that the “community” would provide alternatives. Such meagre response as there has been from local authorities and voluntary bodies has been entirely irrelevant to the most needy’.^110^ In this social and political context, as families were expected to assume the primary responsibility for long-term care of psychiatric patients, the National Schizophrenia Fellowship was deeply concerned that psychiatric research and assumptions around family relations rarely took into account the extreme psychological toll that this form of care took on relatives. As social worker Clare Creer noted in 1978, ‘the strain and tension of living with someone who is so unpredictable, and whose moods can change so suddenly, is hard to imagine for anyone who has not experienced it’.^111^ In the Fellowship’s project ‘Schizophrenia at home’, which aimed to gather family members’ perspectives, relatives often described their situation as ‘living on the edge of a volcano’.^112^ The Fellowship thus produced a wealth of materials aimed at supporting and advising family members who cared for schizophrenic patients, and their engagement with the existing psychiatric research was extremely cautious and often ambivalent.

This was filling an important gap: the Fellowship was founded soon after the publication in 1970 of a letter written by journalist John Pringle, who described how his family was affected when his son was diagnosed with schizophrenia in his twenties. Pringle wrote poignantly and critically about the general lack of systemic support but zoomed in on his family’s experiences of confusion and disorientation when faced with the son’s unusual and difficult-to-read behaviour. According to Pringle, healthcare staff was unable to offer coherent and helpful advice on how the family should behave towards the patient: ‘On almost any specific point on which advice was desperately needed – should he be persuaded to get up, dress, keep himself clean, encouraged to work or study, or just be left alone, which course is best for him? – we grew used to receiving from the doctors’ weary platitudes about showing “patience”.’^113^ In Pringle’s experience, therefore, it was precisely in those areas of emotional behaviour which were deemed vital in EE research that families of schizophrenic patients were left without any meaningful guidance.

In the light of such an extreme vulnerability and glaring lack of support, the National Schizophrenia Fellowship approached the psychiatric research on families with scepticism. It was a morally dubious choice, they argued, to place so much responsibility for the outcome (and onset) of schizophrenia on families while providing them with so little institutional and structural support. Before anything else, the NSF insisted that ‘it is perfectly normal not to be able to cope with these things. Family relationships will be strained to the limit’^114^ – especially in the context of deinstitutionalisation, where the burden of care was bound to fall disproportionately on family members. It naturally distanced itself from the theories of schizophrenogenic mothers or fathers, referring to them as ‘unproven and unhelpful’,^115^ and concluding that ‘family problems are more likely to be the result, and not the cause, of a family member having schizophrenia’.^116^ In some of its earlier literature for relatives, the NSF also argued that, despite close investigations, ‘there [was] no proof’ in the claims that ‘stressful family relations’ played a role in causing or perpetuating schizophrenic symptoms.^117^ Later on, the NSF seemed to accept some of the conclusions coming out of the EE research conducted at the MRC’s Social Psychiatry Unit, and noted that parental behaviour in infancy had nothing to do with causing schizophrenia but highlighted that highly critical behaviour was related to risks of relapse, although it added that ‘such criticism is entirely understandable’.^118^ Families were also advised to avoid emotional overinvolvement, defined as ‘much over-protectiveness and constant anxiety over minor matters to do with the patient’.^119^ However, given that it emphasised minor issues, this definition likely would not include strong (normal?) emotional reactions to highly distressing and disruptive behaviours.

EE scales and insights produced a variety of programmes of psychoeducation and other therapeutic work with (high EE) families to teach them about the role of expressed emotion in aiding or preventing recovery, and to guide them in modelling their behaviour and responses to their ill relatives.^120^ For instance, the London study of intervention in families of schizophrenic patients, which was assessed to have achieved significant positive results, included four core areas of information: diagnosis, symptomatology, course and origins, and treatments. According to the lead researchers, Leff and Vaughn, one of the most important achievements of this intervention design was that it familiarised family members with the diagnosis of schizophrenia and its clinical meaning – in fact, ‘the greatest change that occurred was in the number of high-EE experimental relatives who knew that the diagnosis was schizophrenia after the program’.^121^ This was a surprising result and difficult to understand – many family members seemed to be unaware of why the patients were ill, claiming that they had never been told.^122^ This psychoeducation thus focused on the diagnosis itself – which was in itself surprising, given that DOSMED EE research often praised the emotional atmospheres of developing world families precisely because they distanced themselves from the medical language. In the London study, on the other hand, it was particularly important to explain to family members how the psychiatric profession defined and understood schizophrenia, its origins, causes, and outcomes. The focus was on convincing families that patients held no responsibility for their illness and related behaviour: ‘because the patient cannot usually explain what is happening in his mind, it is not always easy for other people, even those who live with him like yourselves, to realize that many of the odd or upsetting things he does are caused by the illness’.^123^ The education materials repeated several times that patients ‘can’t help all this’ and that upsetting or bizarre behaviours ‘are not done to annoy you’ but were due to both the illness and the medication.^124^ Equally importantly, the materials emphasised that it was possible to recover from schizophrenia, and thereby attempted to counter the overwhelming pessimism that family members reportedly experienced when they encountered the diagnosis. The reported outcome was that high EE family members in particular became more positive when it came to the future and illness prognosis.^125^

It is important to note here that both of these achievements – convincing relatives that patients did not bear any personal responsibility for schizophrenia and that recovery was possible or even likely – seemed to be inspired by what EE research identified as the most significant and widespread ‘traditional’ beliefs in the developing world. As Waxler, Leff, Jenkins, and others argued, outside the West, family members tended to assume that schizophrenia was caused by factors entirely external to the patient, and, since they had little access to the medical language and knowledge, that schizophrenia was likely to be brief and curable. These EE-inspired family interventions thus aimed to produce the same results but – somewhat paradoxically – went about this goal by focusing on the diagnosis itself and its implications. At the same time, the intervention’s educational materials advised relatives ‘not to spend so much time’ with the patient, and ‘not to get too involved’, arguing that independence and autonomy were the most significant factors and that it was often highly beneficial for patients to live in a hostel rather than with parents.^126^ While the materials acknowledged that family members might worry whether they would come across as uncaring, they reassured them that ‘low-EE relatives had more empathy with the patients than high-EE relatives’, although this conclusion was not elaborated any further.^127^

Given that families of schizophrenic patients often reported their experiences of alienation from the mental health profession, it was likely that the development and implementation of possible family intervention designs stemming from EE research would be complicated. Hatfield noted that such intervention programmes rested on problematic assumptions – that it was families who should now be the primary caregivers and take on the burden for treating schizophrenia, while resources were channelled away from hospitals and residential facilities and invested in family training.^128^ This was an important implication of family and EE research, even though it was rarely spelled out. Hatfield speculated that this was mainly because of the highly ‘unrewarding’ nature of psychiatric work with chronic schizophrenia patients, and the fact that psychiatric treatment was extremely likely to fail in those cases.^129^ On the other hand, there was a possibly significant economic argument here that has remained unexplored. In the healthcare system in which families were expected to assume the main role in dealing with schizophrenia, this could lead to large savings if mental health professionals could ensure that patients did not need to return to hospitals due to relapses and that they did not need long-term accommodation in medical or residential facilities for the mentally disturbed. Moreover, the core assumptions around EE levels in developing world families also potentially meant that there was reduced need for investing in mental health facilities in those regions. In that sense, EE-related designs of family interventions, and their prioritisation of families in understanding the course and outcome of schizophrenia, worked directly or indirectly into the hands of the broader liberal ideology of individualising responsibility and minimising investment in social (and healthcare) services. It was no accident that families of schizophrenic patients were highly concerned with the processes of deinstitutionalisation, and regularly critiqued the profound lack of institutional access and support.^130^ While there is no evidence at all that EE researchers consciously bought into this particular political agenda, the broader implications of this entire field of research certainly reinforced the trend of personalising responsibility for mental healthcare, channelling resources away from psychiatric institutions, and focusing on family training and interventions.

## Conclusion

EE research on families of schizophrenic patients has been exceptionally influential. Following DOSMED and its successful internationalisation and translation of the core EE research instruments, hundreds of similar studies have been implemented across the world. Overall, these multiple investigations upheld the general idea that there was an important and meaningful link between the course of schizophrenia and the emotional atmosphere within families. Moreover, global EE research also reinforced the argument that cultural contexts and values might have a major impact on the course and outcome of schizophrenia because they deeply shaped family relations and forms of communication and played an important role in determining whether harmful or beneficial aspects of EE would be dominant in any particular society. These ideas informed a lot of transcultural psychiatric research on schizophrenia since the 1970s and 1980s. DOSMED and the broader field of EE research all contributed to the belief that familial relations, emotional expression, and forms of communication were one of the keys to understanding the most puzzling result of the IPSS: that schizophrenia appeared to have a better outcome and more benign course in the developing world. Perhaps most significantly, this body of research reinforced the idea that there were certain properties of developing world families that seemed to have a positive effect on the outcome of schizophrenia, making relapses less likely and frequent; conversely, this argument also implied that Western families exhibited traits, behaviours, and models of emotional expression which worsened the clinical picture and prognosis of schizophrenia. As this article has demonstrated, however, global schizophrenia research into the role of families rested on a variety of assumptions, which perpetuated colonial interpretive frameworks and continued to revolve around the binary of simplicity (of ‘traditional’ societies in the decolonising world) versus complexity (of Western Europe and North America). This is not to say that it was inherently ‘colonial’ to argue that certain aspects of modern or industrialised life might have shaped family relations and structures in ways that had harmful clinical consequences for schizophrenia patients. In a similar vein, the argument regarding better prognosis of schizophrenia in the developing world need not in itself be problematic. This article has focused on the most influential psychiatric interpretations of the difference in the course and outcome, and on the conceptual frameworks employed in order to understand and explain this important statistical and clinical finding. It was in these interpretations that the leading psychiatric researchers of the time relied heavily on romanticised notions of traditional family relations and on the binary of simplicity versus complexity of societal structures and cultural patterns. At the same time, these discussions raised questions regarding alternative interpretations: While the existing explanatory frameworks were constrained by forms of thinking inherited from the colonial era, could these findings be understood in a different context, one that does not reinforce a hierarchical and simplistic understanding of the Global South? As transcultural psychiatry continues to explore the relationship between culture, modernity, schizophrenia prognosis, and outcome, it will be important to reflect on these alternative possibilities and interrogate critically the existing interpretive frameworks. In any case, the debates and assumptions examined in the article reflected political, social, and cultural concerns that were relevant beyond the psychiatric context: They were closely tied to the psychiatric profession’s efforts to reimagine the relationship between culture and individual psyche and re-think the notions of modernity and ‘primitivism’ which played such an important role in colonial medicine.

## Footnotes

1

Nancy E. Waxler , ‘Is Outcome for Schizophrenia Better in Nonindustrial Societies? The Case of Sri Lanka’, The Journal of Nervous and Mental Disease, 167, 3 (1979), 144–158 438782 10.1097/00005053-197903000-00002.

2

Ian Fallon , Jeffrey Boyd , and Christine McGill , Family Care of Schizophrenia (New York: The Guilford Press, 1984); Edward Dolnick , Madness on the Couch: Blaming the Victim in the Heyday of Psychoanalysis (New York: Simon and Schuster, 2007).

3 Alex Cohen scrutinised this line of argumentation regarding schizophrenia and culture, and critiqued Waxler’s thesis, pointing out that other research indicated family abandonment as a major factor in Sri Lankan psychiatric hospitals. This undermined Waxler’s argument that Sinhalese families were universally supportive of sick people, and questioned the IPSS’s hypothesis regarding the correlation between developing countries and positive schizophrenia outcome ( Alex Cohen , ‘Prognosis for Schizophrenia in the Third World: A Reevaluation of Cross-Cultural Research’, Culture, Medicine and Psychiatry, 16, 1 [1992], 53–75 1537236 10.1007/BF00054439; see also: Alex Cohen et al., ‘Questioning an Axiom: Better Prognosis for Schizophrenia in the Developing World?’, Schizophrenia Bulletin, 34, 2 [2008], 229–4417905787 10.1093/schbul/sbm105PMC2632419; Alex Cohen and Craig Morgan , ‘Estimating the Incidence of Psychosis in Diverse Settings’, Psychological Medicine, 51, 3 [2021], 527–831843029 10.1017/S0033291719003702; Craig Morgan , Alex Cohen , and Tessa Roberts , Psychosis: Global Perspectives [Oxford: Oxford University Press, 2023]).

4 Waxler, *op. cit.* (note 1), 157.

5

Nancy E. Waxler , ‘Culture and Mental Illness: A Social Labelling Perspective,’ The Journal of Nervous and Mental Disease,159 (1974), 379–954436663 10.1097/00005053-197412000-00001.

6
*Ibid.*, 387.

7 IPSS and WHO, ‘Report of the International Pilot Study of Schizophrenia’, WHO: Geneva,1973.

8

Orna Ophir , Schizophrenia: An Unfinished History (London: Polity, 2022); Angela Woods , The Sublime Object of Psychiatry: Schizophrenia in Clinical and Cultural Theory (Oxford: Oxford University Press, 2011); Mary Ellen Wood , Life Writing and Schizophrenia (Leiden: Brill, 2013); Jonathan Metzl , Protest Psychosis: How Schizophrenia Became a Black Disease (Boston: Beacon Press, 2010); Kieran McNally , A Critical History of Schizophrenia (Basingstoke: Palgrave Macmillan, 2016).

9

Julian Leff and Christine Vaughn (eds), Expressed Emotion in Families: Its Significance for Mental Illness (New York: The Guilford Press, 1985); Theodore Lidz and Stephen Fleck , Schizophrenia and the Family (Madison: International Universities Press, 1985); Ian Falloon et al., Family Care of Schizophrenia: A Problem-Solving Approach to the Treatment of Mental Illness (New York: The Guilford Press, 1987).

10

Edward Dolnick , Madness on the Couch: Blaming the Victim in the Heyday of Psychoanalysis (New York: Simon and Schuster, 2007).

11

Andrew Scull , ‘Deinstitutionalization: Cycles of Despair’, The Journal of Mind and Behavior, 11, 3/4 (1990), 301–11; Preethy George et al., ‘Cycles of Reform in the History of Psychosis Treatment in the United States’, Social Science and Medicine – Mental Health, 3 (2023), 1–8 10.1016/j.ssmmh.2023.100205PMC1030276037388405; Despo Kritsotaki , Vicky Long , and Matthew Smith (eds), Deinstitutionalization and After: Postwar Psychiatry in the Western World (Basingstoke: Palgrave Macmillan, 2016).

12 WHO Archives, M4/445/13, ‘Carothers’ Study on Mental Health in Africans’, 1953.

13

John Collin Carothers , ‘A Study of Mental Derangement in Africans, and an Attempt to Explain Its Peculiarities, More Especially in Relation to the African Attitude to Life’, East African Medical Journal, 25 (1948), 197–219 18868013 ; Octave Mannoni , Prospero and Caliban: A Study of the Psychology of Colonisation (London: Methuen & Co, 1956); Megan Vaughan , Curing Their Ills: Colonial Power and African Illness (London: Polity Press, 1991), 101 ; J.F. Ritchie , ‘The African as Suckling and as Adult: A Psychological Study’, Rhodes-Livingstone Papers, 9 (1943), 1–61 .

14

H.B.M. Murphy and B.M. Taumoepeau , ‘Traditionalism and Mental Health in the South Pacific: A Re-Examination of an Old Hypothesis’, Psychological Medicine, 10, 3 (1980), 471–827443902 10.1017/s003329170004736x.

15

Kim Hopper et al. (eds), Recovery from Schizophrenia: An International Perspective: A Report From the WHO Collaborative Project, the International Study of Schizophrenia (Oxford: Oxford University Press, 2007).

16 National Archives Kew, FD12/1273, MRC Social Psychiatry Unit, Progress Report 1979, 9.

17 WHO Archives, M4/445/37 (B), Wig et al., ‘The cross-cultural transfer of ratings of relatives’ expressed emotion’, 4–5.

18 WHO Archives, M4/445/37 (B), ‘The determinants of outcome of severe mental disorder project’.

19

George W. Brown , et al., ‘Influence of Family Life on the Course of Schizophrenic Illness’, British Journal of Preventive and Social Medicine, 16 (1962), 55–68 .

20 WHO Archives, *op. cit.* (note 18).

21

Assen Jablensky et al., ‘Schizophrenia: Manifestations, Incidence and Course in Different Cultures. A World Health Organization Ten-Country Study’, Psychological Medicine Monograph Supplement, 20 (1992), 88–910.1017/s02641801000009041565705.

22 National Archives Kew, FD12/1273, MRC Social Psychiatry Unit, Progress Report 1959–1962, ‘Follow-up studies of discharged psychiatric patients’, 8–9.

23
*Ibid.*; George Brown , ‘The family of the schizophrenic patient’, in A. Coppen and A. Walk (eds), Recent Developments in Schizophrenia: A Symposium (London: Royal Medico-Psychological Association, 1967).

24

George Brown , ‘Experiences of Discharged Chronic Schizophrenic Mental Hospital Patients in Various Types of Living Group’, Millbank Memorial Fund Quarterly, 37 (1959), 105–3113643777.

25 George Brown, ‘The discovery of expressed emotion: Induction or deduction?’, in Julian Leff and Christine Vaughn (eds), *op. cit.* (note 9), 7–25.

26
*Ibid.*, 22.

27 National Archives Kew, FD12/1273, MRC Social Psychiatry Unit, ‘Schizophrenia at home’, 3 December 1975, 10.

28
*Ibid.*

29 Leff and Vaughn, ‘Low-EE versus high-EE relatives: Distinguishing characteristics’, in Leff and Vaughn (eds), *op. cit.* (note 9), 113–20.

30
*Op. cit.* (note 27).

31 WHO Archives, M4/445/37 (B), WHO collaborative study on determinants of outcome of severe mental disorder – sub-study of the relationship between emotional interaction in the family and the short-term prognosis of schizophrenia.

32

Julian Leff , ‘Research on Expressed Emotion: The State of the Art’, International Journal of Mental Health, 24, 2 (1995), 3–6 .

33
*Ibid.*, 4.

34 WHO Archives, M4/445/37 (B), K. Menon et al. ‘Distribution of Expressed Emotion Components among Relatives of Schizophrenic Patients in Aarhus and Chandigarh’, 11.

35 WHO Archives, M4/445/37 (B); Julian Leff, N.N. Wig and A. Gosh, ‘Relatives’ expressed emotion and the course of schizophrenia in Chandigarh: A two-year follow-up of a first contact sample’.

36 Julian Leff and Christine Vaughn, ‘Intervention in families of schizophrenic patients’, in Julian Leff and Christine Vaughn (eds), *op. cit.* (note 9), 132.

37

Julian Leff , ‘Culture and the Differentiation of Emotional States’, British Journal of Psychiatry, 123, 574 (1973), 299–306 10.1192/bjp.123.3.2994746107.

38

N.N. Wig , et al., ‘Expressed Emotion and Schizophrenia in North India: II. Distribution of Expressed Emotion Components among Relatives of Schizophrenic Patients in Aarhus and Chandigarh’, British Journal of Psychiatry, 15, 12 (1987), 165 10.1192/bjp.151.2.1603690104.

39
*Ibid.*

40 WHO Archives, M4/445/37 (B), Leff, Wig and Gosh, ‘Relatives’ expressed emotion and the course of schizophrenia in Chandigarh’, 14.

41
*Ibid.*

42
*Ibid.*

43

J. Leff , et al., ‘Expressed Emotion and Schizophrenia in North India: III. Influence of Relatives’ Expressed Emotion on the Course of Schizophrenia in Chandigarh’, British Journal of Psychiatry, 151, 2 (1987), 166–7310.1192/bjp.151.2.1663690105.

44 WHO Archives, *op. cit.* (note 40), 8–9.

45
*Ibid.*

46

Marvin Karno et al., ‘Expressed Emotion and Schizophrenic Outcome among Mexican-American Families’, Journal of Nervous and Mental Disease, 175, 3 (1987), 143–513819710 10.1097/00005053-198703000-00004.

47

Janis Jenkins and Marvin Karno , ‘The Meaning of Expressed Emotion: Theoretical Issues Raised by Cross-Cultural Research’, American Journal of Psychiatry, 149, 1 (1992), 15 10.1176/ajp.149.1.91728192.

48 Karno et al., *op. cit.* (note 46), 151.

49

Janis Jenkins , ‘Ethnopsychiatric Interpretations of Schizophrenic Illness: The Problem of Nervios Within Mexican-American Families’, Culture, Medicine and Psychiatry, 12 (1988), 305 10.1007/BF000519723234015.

50 Karno et al., *op. cit.* (note 46), 151.

51

Janis Jenkins , ‘Conceptions of Schizophrenia as a Problem of Nerves: A Cross-Cultural Comparison of Mexican-Americans and Anglo-Americans’, Social Science and Medicine, 26, 12 (1988), 1233–433206245 10.1016/0277-9536(88)90155-4.

52
*Ibid.*, 1240–41.

53
*Ibid.*; Jenkins, *op. cit.* (note 49), 310–20.

54
*Ibid.*, 1241.

55
*Ibid.*

56
*Ibid.*

57
*Ibid.*, 1240–1.

58 Jenkins and Karno, *op. cit.* (note 47), 9–21.

59

H.B.M. Murphy , ‘Cultural Factors in the Genesis of Schizophrenia’, Journal of Psychiatric Research, 6, 11 (1968), 137–153 .

60 Brown, *op. cit.* (note 25), 22.

61
*Ibid.*

62 George Brown et al., *op. cit.* (note 19), 55.

63
*Ibid.*, 55–68.

64

Vaughn and Leff , ‘The Influence of Family and Social Factors on the Course of Psychiatric Illness’, British Journal of Psychiatry, 129 (1976), 135 10.1192/bjp.129.2.125963348.

65

Vaughn and Leff , ‘Patterns of Emotional Response in Relatives of Schizophrenic Patients’, Schizophrenia Bulletin, 7, 1 (1981), 43–47233111 10.1093/schbul/7.1.43.

66 Brown et al., *op. cit.* (note 62), 56.

67

Trude Tietze , ‘A Study of Mothers of Schizophrenic Patients’, Psychiatry, 21, 1 (1949), 55–65 10.1080/00332747.1949.1102271618119648.

68 Brown et al., *op. cit.* (note 62), 66–8.

69

Carol E. Hartwell , ‘The Schizophrenogenic Mother Concept in American Psychiatry’, Psychiatry, 59, 3 (1996), 274–978912946 10.1080/00332747.1996.11024768; Elaine Showalter , The Female Malady: Women, Madness and English Culture, 1830–1980 (London: Virago Press, 1987).

70

John N. Rosen , Direct Analysis: Selected Papers (New York: Grune and Stratton, 1953), 8–9 .

71

Theodore Lidz et al., ‘The mothers of schizophrenic patients’, in Theodore Lidz, S. Fleck , and A.R. Cornelison (eds), Schizophrenia and the Family (Madison: International Universities Press, 1985), 76–7.

72

Theodore Lidz , ‘Schizophrenia, human integration and the role of the family’, in D.D. Jackson (ed.), The Etiology of Schizophrenia (New York: Basic Books, 1960), 338 .

73

David M. Levy , ‘Maternal Overprotection and Rejection’, Archives of Neurology and Psychiatry, 25 (1931), 886–9.

74 Hartwell, *op. cit.* (note 69), 282.

75 Leff and Vaughn, ‘Expressed emotion and the etiology of schizophrenia’, in Leff and Vaughn (eds), *op. cit.* (note 9), 220.

76 Showalter, *op. cit.* (note 69), 220–47.

77
*Ibid.*, 222.

78
*Ibid.*

79 Leff and Vaughn, *op. cit.* (note 75), 224.

80

D.F. Ricks and C. Nameche , ‘Symbiosis, Sacrifice and Schizophrenia’, Mental Hygiene, 50 (1966), 540–515969624; M. Waring and D. Ricks , ‘Faily Patterns of Children Who Become Adult Schizophrenics’, Journal of Nervous and Mental Diseases, 140 (1965), 351–6410.1097/00005053-196505000-0000414334256.

81 Leff and Vaughn, *op. cit.* (note 75), 217–8.

82

Vaughn et al., ‘Family Factors in Schizophrenic Relapse’, Archives of General Psychiatry, 8, 2 (1982), 425–6.

83
*Ibid.*

84

Leff et al., ‘A Controlled Trial of Social Intervention in the Families of Schizophrenic Patients’, British Journal of Psychiatry,141 (1982), 121–3410.1192/bjp.141.2.1217116051.

85
*Ibid.*

86
*Ibid.*, 119.

87 Leff and Vaughn. ‘Low EE versus high EE relatives: Distinguishing characteristics’, in Leff and Vaughn (eds), *op. cit.* (note 9), 117.

88
*Ibid.*, 118.

89 Leff and Vaughn, ‘The expressed emotion scales’, in Leff and Vaughn (eds), *op. cit.* (note 9), 47–8.

90
*Ibid.*, 49.

91
*Ibid.*, 49–50.

92
*Ibid.*, 50–2.

93
*Ibid.*, 48.

94
*Ibid.*, 48–9.

95
*Ibid.*, 49.

96
*Ibid.*, 51.

97 Leff and Vaughn. ‘Low EE versus high EE relatives: Distinguishing characteristics’, in Leff and Vaughn (eds), *op. cit.* (note 9), 118–9.

98
*Ibid.*, 118.

99

Agnes Hatfield et al., ‘Expressed Emotion: A Family Perspective’, Schizophrenia Bulletin, 13, 2 (1987), 221–63616514 10.1093/schbul/13.2.221.

100 Vaughn and Leff, *op. cit.* (note 65), 44.

101 WHO Archives, M4/445/37 (B), ‘Relatives’ expressed emotion and the course of schizophrenia in Chandigarh: A two-year follow-up of a first contact sample’, 7–9.

102 Leff et al., *op. cit.* (note 43), 169.

103 Wig et al., *op. cit.* (note 38), 163.

104
*Ibid.*, 165.

105
*Ibid.*

106 Hatfield et al., *op-cit.* (note 99), 225.

107
*Ibid.*, 222–3.

108
*Ibid.*, 222.

109

Andrew Scull , ‘“Community Care”: Historical Perspective on Deinstitutionalization’, Perspectives in Biology and Medicine, 64, 1 (2021), 70–81 33746131 10.1353/pbm.2021.0006.

110 Wellcome Library, Archives and Manuscripts, PP/DSC/C/1/8, ‘Why the NSF?’, 7.

111

Clare Creer , ‘Social work with patients and their families’, in John Wing (ed.), Schizophrenia: Towards a New Synthesis (London: Academic Press, 1978), 240–1.

112
*Ibid.*

113 John Pringle, ‘A case of schizophrenia’, *The Times*, 9 May 1970.

114
*Wellcome Library, Archives and Manuscripts*, PP/DSI/C/1/17, ‘About schizophrenia’, 12.

115
*Ibid.*, PP/DSI/C/1/17, NSF, ‘About schizophrenia’, 7.

116
*Ibid.*, ‘If you are caring for someone with schizophrenia, you are not alone’.

117
*Ibid.*, ‘Schizophrenia’, 1980, 7.

118
*Ibid.*, ‘Schizophrenia’, 1985, 5.

119
*Ibid.*, ‘About schizophrenia’, 12–16.

120 Leff and Vaughn, *op. cit.* (note 36), 123–49.

121
*Ibid.*, 131.

122 The researchers concluded they must have been shocked when they received the diagnosis and remained in denial (*ibid.*, 131). However, the NSF reported how difficult it was to get the firm diagnosis of schizophrenia, especially in the case of young patients, whose families were often told for years that their child suffered from neurosis or depression (Wellcome Library, Archives and manuscripts, PP/DSI/C/1/3, ‘Schizophrenia: The family burden’).

123 Leff and Vaughn, *op. cit.* (note 36), Appendix: Education materials, 144.

124
*Ibid.*, 145–7.

125
*Ibid.*, 147–9.

126
*Ibid.*, 148–9.

127
*Ibid.*, 133.

128 Hatfield et al., *op. cit.* (note 99), 224–5.

129
*Ibid.*

130
*Ibid.*

